# Artificial Intelligence for the Detection of Diabetic Retinopathy in Primary Care: Protocol for Algorithm Development

**DOI:** 10.2196/12539

**Published:** 2019-02-01

**Authors:** Josep Vidal-Alaball, Dídac Royo Fibla, Miguel A Zapata, Francesc X Marin-Gomez, Oscar Solans Fernandez

**Affiliations:** 1 Health Promotion in Rural Areas Research Group Gerència Territorial de la Catalunya Central Catalan Health Institute Sant Fruitós de Bages Spain; 2 Unitat de Suport a la Recerca de la Catalunya Central Institut Universitari d’Investigació en Atenció Primària Jordi Gol Sant Fruitós de Bages Spain; 3 OPTretina Sant Cugat del Vallès Spain; 4 eHealth Directorate Department of Health Government of Catalonia Barcelona Spain

**Keywords:** diabetes mellitus, diabetic retinopathy, fundus oculi, artificial intelligence, computer assisted diagnosis, neural network computer

## Abstract

**Background:**

Diabetic retinopathy (DR) is one of the most important causes of blindness worldwide, especially in developed countries. In diabetic patients, periodic examination of the back of the eye using a nonmydriatic camera has been widely demonstrated to be an effective system to control and prevent the onset of DR. Convolutional neural networks have been used to detect DR, achieving very high sensitivities and specificities.

**Objective:**

The objective of this is paper was to develop an artificial intelligence (AI) algorithm for the detection of signs of DR in diabetic patients and to scientifically validate the algorithm to be used as a screening tool in primary care.

**Methods:**

Under this project, 2 studies will be conducted in a concomitant way: (1) Development of an algorithm with AI to detect signs of DR in patients with diabetes and (2) A prospective study comparing the diagnostic capacity of the AI algorithm with respect to the actual system of family physicians evaluating the images. The standard reference to compare with will be a blinded double reading conducted by retina specialists. For the development of the AI algorithm, different iterations and workouts will be performed on the same set of data. Before starting each new workout, the strategy of dividing the set date into 2 groups will be used randomly. A group with 80% of the images will be used during the training (training dataset), and the remaining 20% images will be used to validate the results (validation dataset) of each cycle (epoch). During the prospective study, true-positive, true-negative, false-positive, and false-negative values will be calculated again. From here, we will obtain the resulting confusion matrix and other indicators to measure the performance of the algorithm.

**Results:**

Cession of the images began at the end of 2018. The development of the AI algorithm is calculated to last about 3 to 4 months. Inclusion of patients in the cohort will start in early 2019 and is expected to last 3 to 4 months. Preliminary results are expected to be published by the end of 2019.

**Conclusions:**

The study will allow the development of an algorithm based on AI that can demonstrate an equal or superior performance, and that constitutes a complement or an alternative, to the current screening of DR in diabetic patients.

**International Registered Report Identifier (IRRID):**

PRR1-10.2196/12539

## Introduction

Diabetic retinopathy (DR) is one of the most important causes of blindness worldwide, especially in the most developed countries [1,2]. Up to 20% of type 2 diabetics have DR lesions at the time of diagnosis, and after 20 years of evolution of the illness, >60% of the patients have developed DR. The percentage of diabetic patients who have never undergone an ophthalmoscopic exploration exceeds 30% according to different studies [3].

DR appears and evolves asymptomatically for years, and it is in the early stages (asymptomatic) when the treatments to avoid vision loss are really effective. With early detection, DR can be treated with techniques that have been shown to reduce the risk of severe vision loss by >90% [3].

Regularly examining the fundus of the eye of known diabetic patients using a nonmydriatic camera has been widely shown to be an effective system to control and prevent the onset of DR [3-6]. Nonmydriatic retinal photography is a good alternative to direct ophthalmoscopy for the screening of DR; it offers high sensitivity and specificity (87% and 97%, respectively), simplicity of the technique, greater accessibility, ease in the registration of information (the computerized file that allows the evolutionary monitoring of the lesions), and better cost-effectiveness ratio compared with the ophthalmoscopy method with pupillary dilatation [7,8].

On the other hand, in recent years, there has been a substantial improvement in the field of artificial intelligence (AI) applied to the classification of medical images through deep learning techniques using convolutional neural networks (CNNs) [9]. In some cases, performances comparable to those obtained using specialist physicians have been reported [10-12]. These CNNs have also been used for the detection of DR, obtaining high sensitivities and specificities [13,14] with accuracies of up to 97.71% [15,16]. A recent study has reported a sensitivity and specificity of 92.5% and 98.5%, respectively. In this study, 85.6% of false-positive cases were due to a misclassification of mild or moderate DR and 77.3% of all false-negative cases occurred for undetected intraretinal microvascular abnormalities [17]. However, none of these algorithms have been developed with a population from southern Europe.

The current state-of-the-art screening for AI systems for medical images like the fundus images is a combination of AI technology (deep learning system) connected to a reading center with a board of retinal experts to confirm the positive cases diagnosed by the deep learning system and optimized to achieve high sensitivities. An AI system incorporated into routine clinical practice to detect DR is currently being beta-tested by the Singapore National Diabetic Retinopathy Screening Program [18,19].

The aim of this study is to develop an AI algorithm for the detection of signs of DR in diabetic patients and to scientifically validate the algorithm to be used as a screening tool in primary care.

## Methods

### Study Design

This project will follow a methodology similar to that used by Li et al [17] and will consist of 2 concomitant studies: In the first study, we will develop an AI algorithm to detect the signs of DR in patients with diabetes. The phases of the study are described in Textbox 1.

The second part of the project will consist of the elaboration of a prospective study that will allow comparing the diagnostic capacity of the algorithm with that of the family medicine physicians and with retina specialists. The reference will be a blinded double reading conducted by the retina specialists (with a blinded third reading in case of disagreement in the previous 2 readings). In this way, the results obtained, both by the AI algorithm and by family medicine specialists, will be compared using the gold standard (accuracy, sensitivity, specificity, area under the curve, etc). The inclusion of nurses who received training in fundus readings will be considered to compare their diagnostic capacity.

### Study Population, Site Participation, and Recruitment

Images for the development of the algorithm will be ceded by the CHS and will include images from the whole Catalan population. The prospective study will take place in the primary care centers managed by the Catalan Health Institute in Central Catalonia, which includes the counties of Bages, Osona, Berguedà, and Anoia. The reference population will be the population assigned to these primary care centers. This population included about 512,000 people in 2017 [20], with an estimated prevalence of diabetes of 7.1% [21].

The study period will include 2010-2017 for the development of the algorithm with AI. The prospective study will begin once the algorithm is developed and will run until the number of readings needed is obtained (about 3-4 months).

### Conduct of the Study

For the development of the AI algorithm, all fundus images labeled as DR of patients from primary care centers in Catalonia between 2010 and 2017 will be included. For the prospective study, all the images of patients who underwent an eye fundus examination will be included from the study start period until the adequate number of patients is reached.

A high percentage of fundus images must have sufficient quality; that is, a 40-degree vision of the central retina where at least a three-fourth part of the optic nerve, a well-focused macula, and well-defined veins and arteries of the upper and lower arcs can be seen. Eye fundus images that do not have adequate technical quality (dark) or that cannot be evaluated due to the opacity of the media (eg, for cataracts) will be excluded. Development of the AI algorithm is explained in Textbox 2.

**Phases of the first study.**

1. Transfer of anonymized retinal images labeled as DR by the Department of Health though the Catalan Health Service (CHS).
2. Evaluation of the quality of the images to discard images of very low quality and evaluation of data distribution.
3. Machine learning. Iterative process with 2 phases (training and adjustments) until satisfactory results are obtained:
  - Training of the machine with the dataset and obtention of results.
  - Making the necessary adjustments:
    - A specialized engineer from OPTretina will evaluate the possibility of improving the algorithm and will determine the following:
      - The adjustments that should be made in the design of the neural network (preprocessing, number of layers, optimizer, learning rate, dropout, batch size, epoch number, etc) that can help improve the algorithm.
      - The most interesting batch of images that must be revised in order to significantly improve the learning of the algorithm in the next training. These images are images with predictions contrary to labeling (possible mislabeling) and predictions of low confidence (border cases).
    - Retina specialists (collaborators of OPTretina) will review the labeling of all the images selected in the previous step. In the final phases, up to 3 readings from different retina ophthalmologists may be necessary to reach a consensus in the labeling of border cases.
  - Development or installation of the algorithm in the CHS Electronic Medical Records system to be used by family medicine physicians in their workplace in real time. This integration is not essential for the realization of the project, but it will start during the development of the project.

**Development of the artificial intelligence algorithm.**

Independent variables:

- (number of) true positives (TPs)
- (number of) true negatives (TNs)
- (number of) false positives (FPs)
- (number of) false negatives (FNs)

Dependent variables:

- Sensitivity or true positive rate=TP/(TP+FN)
- Specificity or true negative rate=TN/(TN+FP)
- Accuracy=(TP+TN)/(TP+FP+FN+TN)
- Area under the receiver operating characteristic curve: graphic representation that shows the diagnostic capacity of a binary classifier based on the variation in the discrimination threshold. It is obtained by plotting the sensitivity against (1−specificity) under different discrimination thresholds.

### Data Collection

For the development of the AI algorithm, it is necessary to have the anonymized images with the corresponding label that classifies each image (in one of the classes with which the algorithm is to be trained). The personnel responsible for information technology (IT) of the CHS will evaluate the best strategy for the anonymization and extraction of the images from the computer systems of the CHS, as well as the identification of each image with a unique identifier. On the other hand, a tabulated file type CSV or TXT will be required to relate each image identifier with the corresponding classification. The person responsible for IT of the CHS, together with the technical manager of OPTretina, will agree on the best way to transfer these 2 sources of information, in a secure way, from the CHS servers to the OPTretina servers (SSH File Transfer Protocol, external hard disk) depending on the volume of data to be transferred and the internal policy of the CHS. OPTretina is experienced in developing AI models for automatic fundus image classification and is a Spanish Agency of Medicines and Health Products-certified medical device manufacturer.

For the prospective study, anonymized weekly fundus data readings collected by family medicine physician readers of fundus images in Central Catalonia will be collected. The images will be transferred to the OPTretina servers to be first analyzed by the diagnostic algorithm and then by the retina specialists who will make the definitive diagnosis. The person responsible for IT of the CHS, together with the technical manager of OPTretina, will agree on the best way to transfer these data in a secure manner.

### Ethical Considerations

We will follow the ethical principles of the Declaration of Helsinki of 1964 reviewed by the World Health Organization in the year 2000 in Edinburgh as well as the Spanish Organic Law 15/1999 Protection of Personal Data of Character adapted to the General Regulation of Data Protection. All information collected will be treated confidentially in strict compliance with legislation in observational studies.

For the development of the AI algorithm, only anonymized data will be used to guarantee at all times the confidentiality of the data shared with the computer systems of OPTretina. Image property rights will always remain with the CHS. OPTretina will return the images once the algorithm has been developed.

Our study does not foresee any contact with patients during the development of the AI algorithm. During the prospective study, family medicine physicians, who are the regular readers of fundus images, will not know the determination made by the algorithm. In this way, the medical criteria of the family doctor will be the usual, without any possibility of interference or bias. During the study, all readings will be blind and independent. At the end of the study, the results of the evaluations of the images of the prospective study will be compared with the readings made by the retina specialists (considered the gold standard) and analyzed. If any discrepancy is detected that is potentially dangerous for the patient, the family doctors who have made the assessment will be informed so that they can take the measures they consider appropriate according to their clinical criteria. This study protocol has been already approved by the Catalan Institute in Primary Care Research (IDIAP Jordi Gol) Health Care Ethics Committee on 29/06/2018 (code P18/109).

### Statistical Analysis

#### Sample Size Calculation

For the development of the AI algorithm, it is convenient to have at least 80,000 fundus images with a distribution of classes (classification groups) that have enough examples of each class. It is recommended that the classes are as balanced as possible and that the minority group has at least 5000 examples. These calculations have been made taking into account the available literature [19] and the conclusions and consensus of specialized discussion groups such as Kaggle [22], among others.

For the prospective study, we calculated that 1000 consecutive patients (who meet the inclusion criteria) would be needed. This number has been calculated taking into account the recent precedent of scientific evidence accepted by the Food and Drug Administration in the validation of a similar algorithm [14,23].

#### Planned Analysis

When developing the AI algorithm, as explained in the methodology and design section, different iterations and trainings will be conducted on the same dataset. Before starting every new training, we will use a widely known strategy in CNN, whereby the dataset is started in 2 groups in a random way. A group with 80% of the images will be used during training (training dataset), and the other, the remaining 20% of the images (validation dataset), will be used to validate the results of each cycle (epoch). Provided we have a large dataset, 80% of instances will be enough to avoid variance in parameter estimation. Using the other 20% for cross-validation will be enough to avoid variance in the performance metric. Depending on the results of the first experiments (training and validation), we will adjust the 80:20 split ratio. At the end of each epoch, we will record the values of accuracy and loss, both for the training dataset and the validation dataset, and will paint a graphic showing the evolution. Analyzing these graphs, the engineer will be able to extract very valuable information to know how many epochs will be necessary, whether the learning rate is adequate, whether the phenomenon known as overfitting is appearing, etc.

With the validation dataset, we will calculate true positives (TPs), true negatives (TNs), false positives (FPs), and false negatives (FNs); from there, we will obtain the confusion matrix as well as the rest of the indicators that measure the performance of the algorithm. With a more detailed image-by-image analysis, the candidate images to be revised in order to improve the quality of the labeling will be obtained.

During the prospective study, family medicine physician readers will evaluate the fundus images as usual and report their findings in the electronic medical notes. After this, they will upload the images, together with a unique patient ID, in a Web application provided by OPTretina. The uploaded images will then be available for the AI algorithm and for the board of retina specialists to perform the corresponding diagnostic and classification analysis. A blinded double reading will be done by the retina specialists with a third reading in case of disagreement.

Once all the patients included have been evaluated, all data will be exported and linked based on the patient unique ID to analyze the results and calculate the performance metrics for the comparisons. Furthermore, we will measure the performance of the AI algorithm using the public Messidor-2 dataset (collection of DR examinations). We will again calculate the values of TP, TN, FP, and FN; from there, we will obtain the confusion matrix and the rest of the indicators to measure the performance of the algorithm. Both the algorithm and the readings made by the team of family medicine physician readers will be compared with the reference blinded double readings made by the retina specialists, and the final indicators will be obtained.

- Sensitivity or true positive rate=TP/(TP+FN)
- Specificity or true negative rate=TN/(TN+FP)
- Accuracy=(TP+TN)/(TP+FP+FN+TN)
- Area under the receiver operating characteristic curve (AUC)

In cases where the AUC of the algorithm is superior to that of the specialists in family medicine readers and superior to 0.75, we will be able to say that we have obtained a good algorithm. The following intervals have been established for different values of AUC [24]:

- [0.5, 0.6]: Bad test
- [0.6, 0.75]: Regular test
- [0.75, 0.9]: Good test
- [0.9, 0.97]: Very good test
- [0.97, 1]: Excellent test

## Results

Cession of the images began at the end of 2018. Once the quality of the images has been evaluated, we will start with the development of the algorithm, which is calculated to last about 2 months. The inclusion of patients in the cohort will begin in early 2019 and is expected to last 3 to 4 months. We expect the preliminary results to be published by the end of 2019 and complete analysis to be published by 2020.

## Discussion

### Summary

This project offers several benefits. First, it facilitates the use of information and knowledge accumulated in the existing database available to the CHS and presentation of a success case of great relevance for similar future projects. Second, other signs of pathology are also detectable in retinal images, which opens the door for the development of new algorithms, such as those for the detection of macular degeneration associated with age, for suspicion of glaucoma, for presence of nevus and epiretinal membrane. This may allow, with certain indications for use, establishment of protocols for screening of general population or of certain risk groups. Third, so far, no similar algorithm has been developed with a population from southern Europe. It would be the first time that images taken from local population from this area are used, giving greater sensitivities and specificities to our environment.

If the results are found to be satisfactory, they can be used as a tool to support family medicine physicians’ decisions and, therefore, can save them valuable time. In addition, if the results of the scientific validation are found to be satisfactory, it will be possible to obtain the CE mark as a sanitary product, which opens the door for its use as an automatic system that does not require the intervention of a doctor.

### Strengths and Limitations of the Study

The difficulties and limitations that we can expect for this project are those related to projects of these characteristics:

- Data volume: It is always difficult to transfer and store gigabytes of images. We will solve these difficulties by hiring Amazon Web Services to obtain the bandwidth and storage capacity necessary to host the data in a secure and encrypted manner.
- Necessary graphic processing capacity: The iterative training of deep neural networks imposes a very important cost in time and money, requiring special servers with a graphic calculator capacity of last generation. To mitigate this limitation, in the preprocessing of the images, the resolution of the images is reduced (eg, from 2400×2400 pixels to 512×512 pixels), which can cause information loss. For example, small microaneurysms (characteristic of incipient DR) cannot be detected in low resolution. With the available bibliography and with the publications of the winners of the Kaggle [22] contest, we know that with 512×512 pixels, we can obtain the best results while at the same time overcoming or adequately mitigating the limitation of the processing capacity necessary during the iterations in learning.
- Presence of noise (problematic images or incorrect labels) that makes learning difficult: A certain level of noise has been shown to be positive in order to obtain a more tolerant and robust algorithm, in view of the real day-to-day data, but it is necessary that the noise ratio is low so that this does not to interfere with the learning of the machine. The noise comes mainly from the following:
  - mislabeled images
  - low-quality images (darkness, brightness, contrast, too much flash, etc)
  - presence of artifacts (dirt on the camera lens)
- Class distribution: Usually, there are many normal images and very few of a certain class or group of pathology. This is one of the main problems presented in the Kaggle contest [22]. In our study, we will not have this problem because we have access to many images labeled with different grades of DR.

### Conclusions

It is possible to develop an algorithm based on AI that can demonstrate an equal or superior performance (measurable and comparable) and that constitutes a complement or an alternative to the current system based on screening of DR performed by family medicine physicians.

## Abbreviations

AI: artificial intelligence
AUC: area under the receiver operating characteristic curve
CHS: Catalan Health Service
CNN: convolutional neural network
DR: diabetic retinopathy
FP: false positive
FN: false negative
IT: information technology
TP: true positive
TN: true negative
